# Supplementary material for: Metabolomic Analysis of the Effect of Freezing on Leaves of Malus sieversii (Ledeb.) M.Roem. Histoculture Seedlings
Source: Int J Mol Sci. 2023 Dec 25;25(1):310. doi: 10.3390/ijms25010310 (PMC10778857; doi:10.3390/ijms25010310)
Supplement: Supplementary file 1 [file ijms-25-00310-s001.zip › Figure S1/Figure legends.docx]

Note: Horizontal coordinates indicate the predicted principal components, and the horizontal direction shows the gap between groups; vertical coordinates indicate the orthogonal principal components, and the vertical direction shows the gap within groups; and the percentage indicates the explanation rate of this component for the dataset. Each point in the graph indicates a sample, samples in the same group are indicated using the same colour, and Group is the grouped.
