# Supplementary material for: Metabolomic Analysis of the Effect of Freezing on Leaves of Malus sieversii (Ledeb.) M.Roem. Histoculture Seedlings
Source: Int J Mol Sci. 2023 Dec 25;25(1):310. doi: 10.3390/ijms25010310 (PMC10778857; doi:10.3390/ijms25010310)
Supplement: Supplementary file 1 [file ijms-25-00310-s001.zip › Figure S2/Figure legends.docx]

Note: The horizontal coordinates represent the model R2Y, Q2 values, and the vertical coordinates are the frequency of occurrence of the model classification effect in 200 random permutation experiments. The orange colour in the figure represents the random permutation model R2Y, the purple colour represents the random permutation model Q2, and the values represented by the black arrows are the R2X, R2Y and Q2 values of the original model.
