# Supplementary material for: Metabolomic Analysis of the Effect of Freezing on Leaves of Malus sieversii (Ledeb.) M.Roem. Histoculture Seedlings
Source: Int J Mol Sci. 2023 Dec 25;25(1):310. doi: 10.3390/ijms25010310 (PMC10778857; doi:10.3390/ijms25010310)
Supplement: Supplementary file 1 [file ijms-25-00310-s001.zip › Figure S3/Figure legends.docx]

Note:The horizontal coordinates indicate the covariance of the principal components with the metabolites, and the vertical coordinates indicate the correlation coefficients between the principal components and the metabolites, the closer the metabolites are to the upper right and lower left corners indicate that the differences are more significant, the red dots indicate that the VIP values of these metabolites are greater than or equal to 1, and the green dots indicate that the VIP values of these metabolites are less than 1.
