# Supplementary figures and images for: Metabolomic Analysis of the Effect of Freezing on Leaves of Malus sieversii (Ledeb.) M.Roem. Histoculture Seedlings
Source: Int J Mol Sci. 2023 Dec 25;25(1):310. doi: 10.3390/ijms25010310 (PMC10778857; doi:10.3390/ijms25010310)

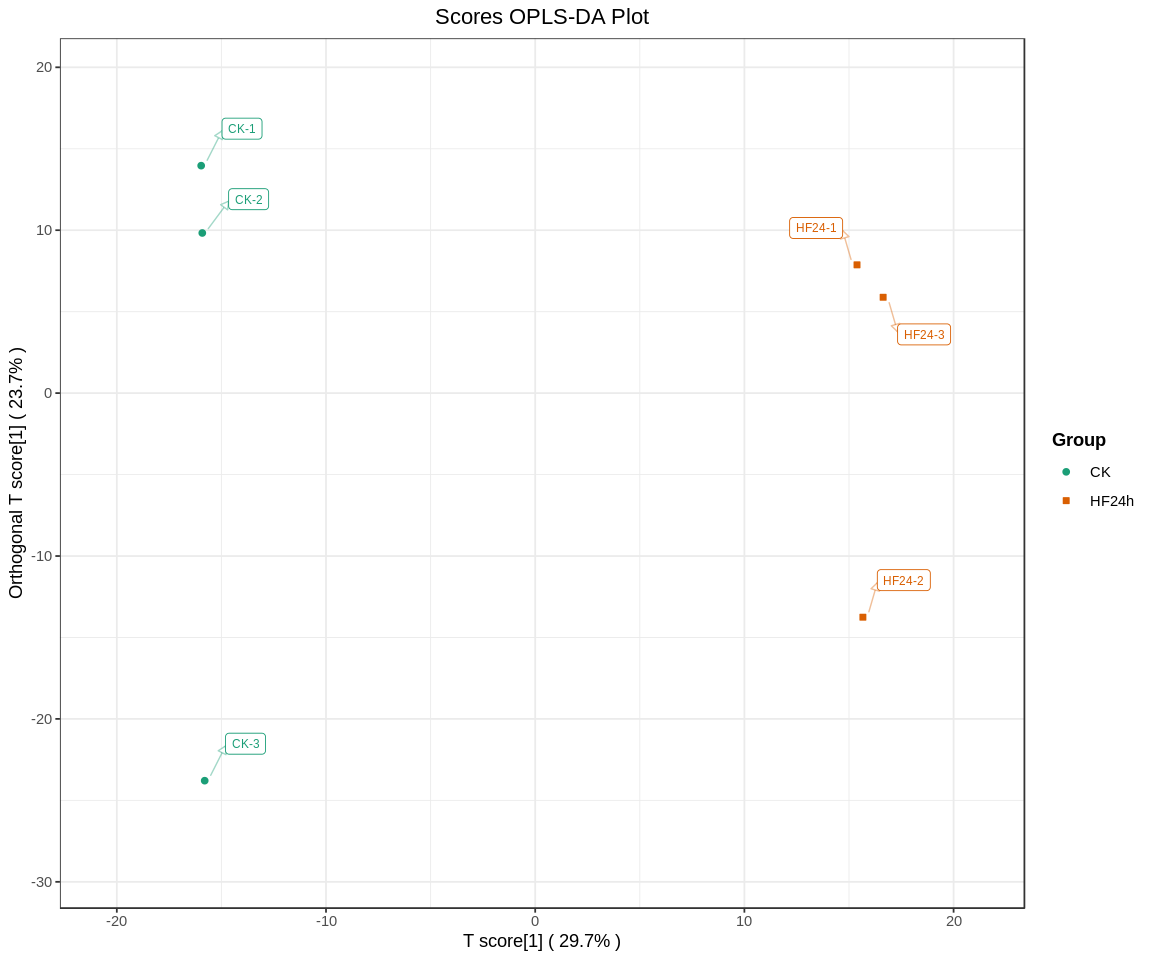

Supplement: Supplementary file 1 [file ijms-25-00310-s001.zip › Figure S1/CKvsHF24H.png]

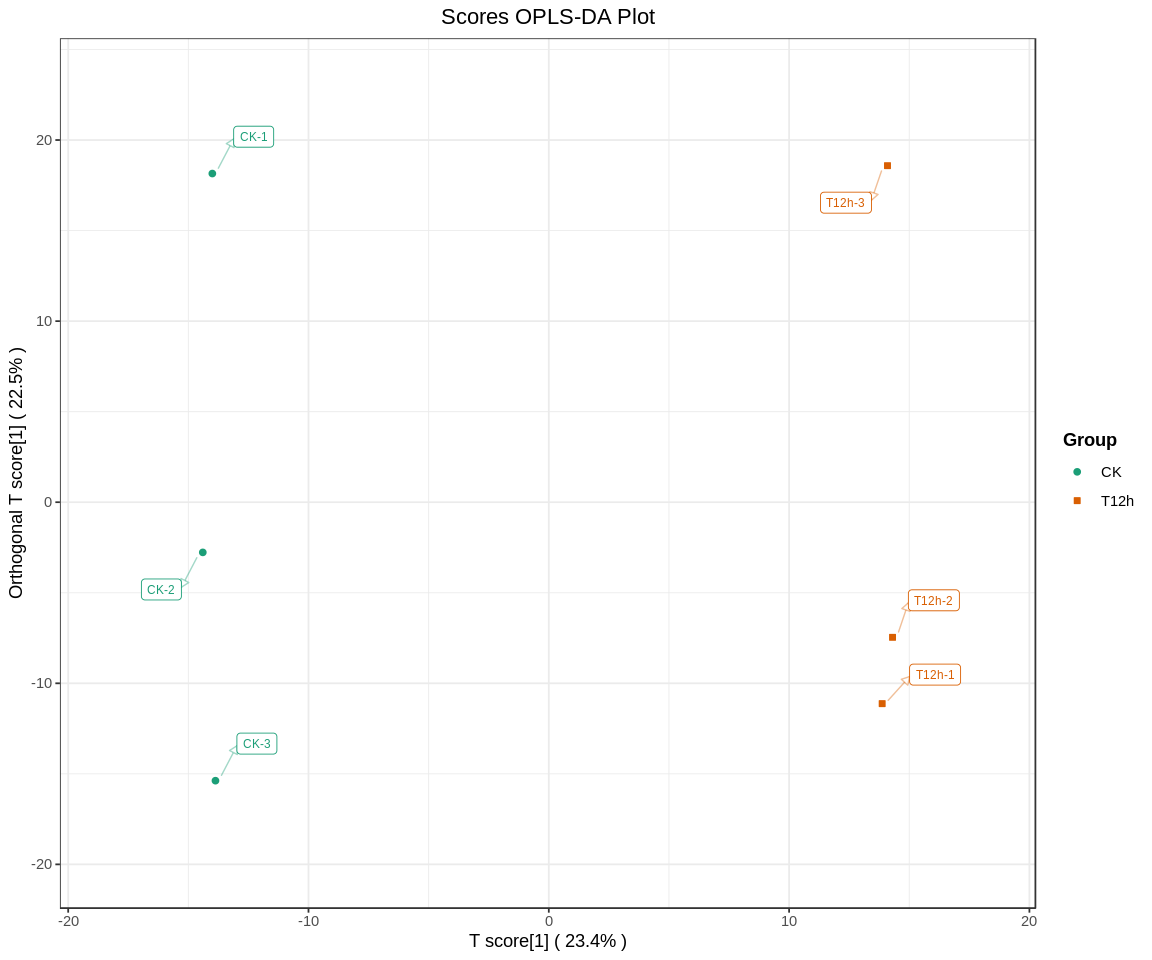

Supplement: Supplementary file 1 [file ijms-25-00310-s001.zip › Figure S1/CKvsT12H.png]

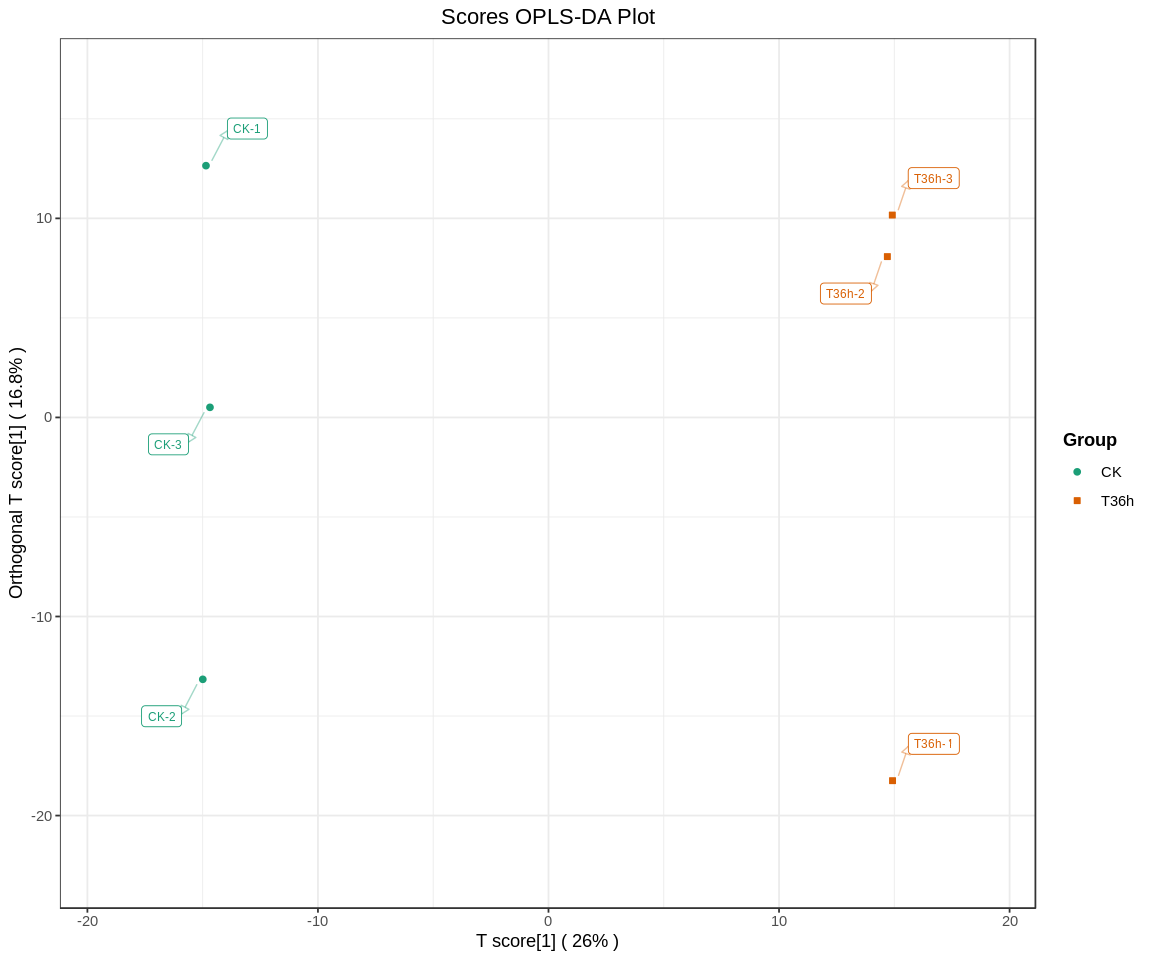

Supplement: Supplementary file 1 [file ijms-25-00310-s001.zip › Figure S1/CKvsT36H.png]

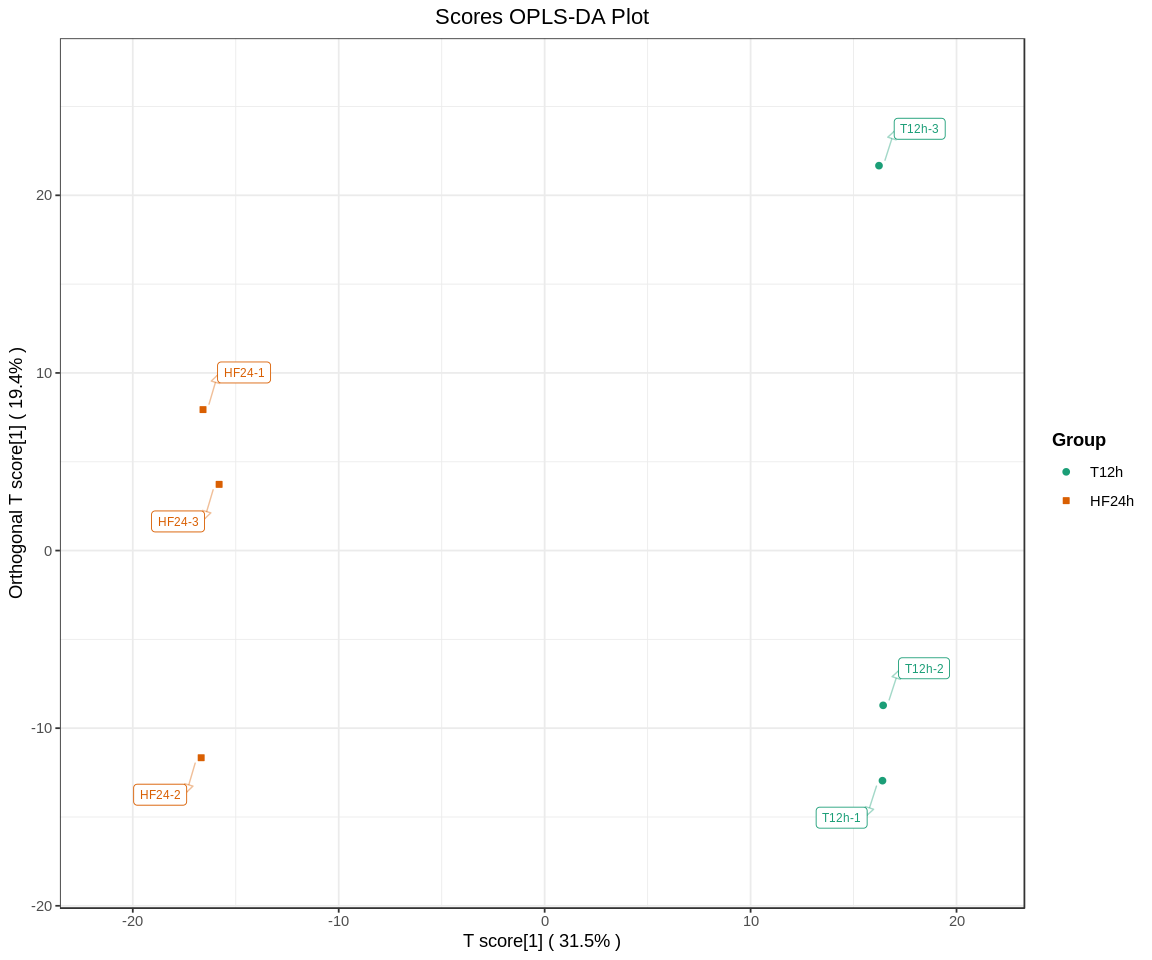

Supplement: Supplementary file 1 [file ijms-25-00310-s001.zip › Figure S1/T12hvsHF24h.png]

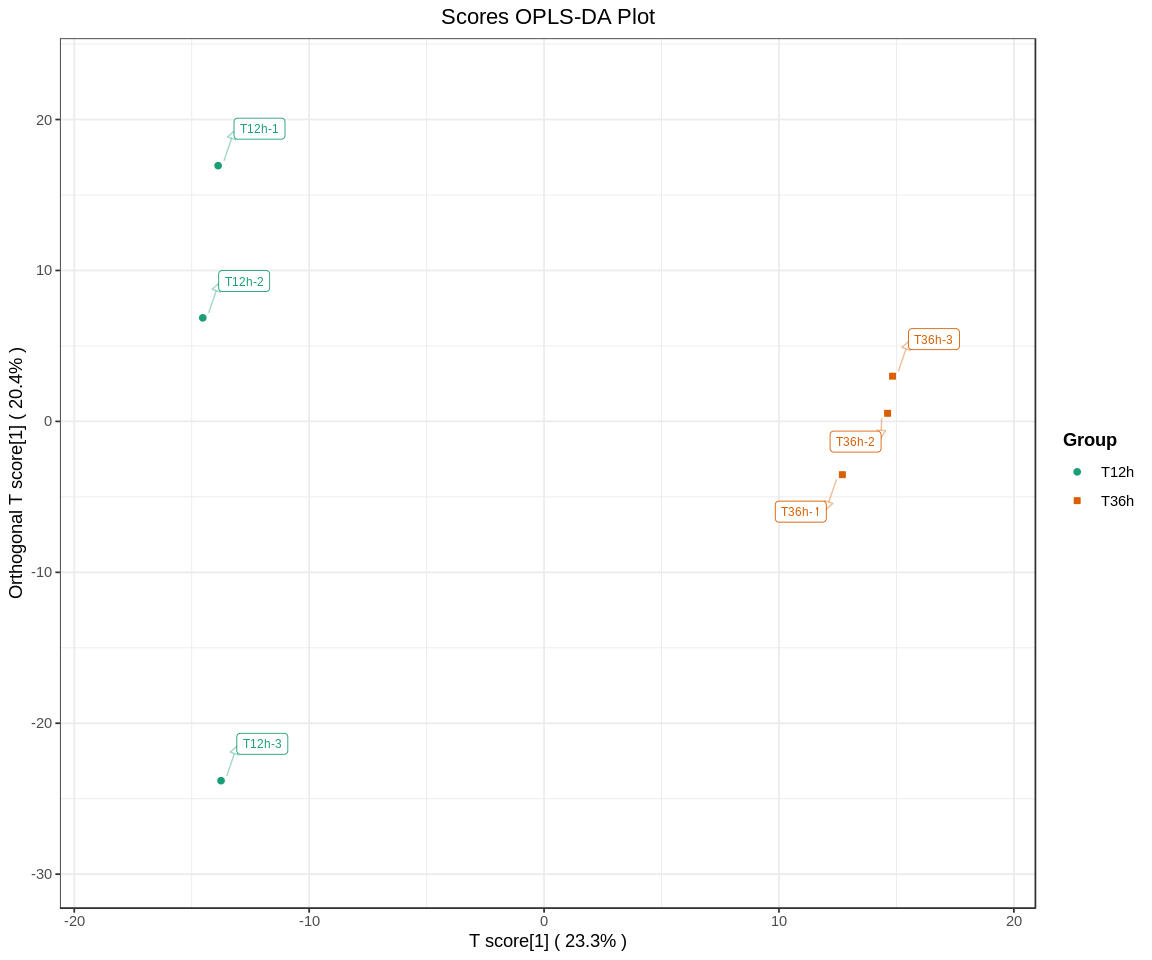

Supplement: Supplementary file 1 [file ijms-25-00310-s001.zip › Figure S1/T12hvsT36h.png]

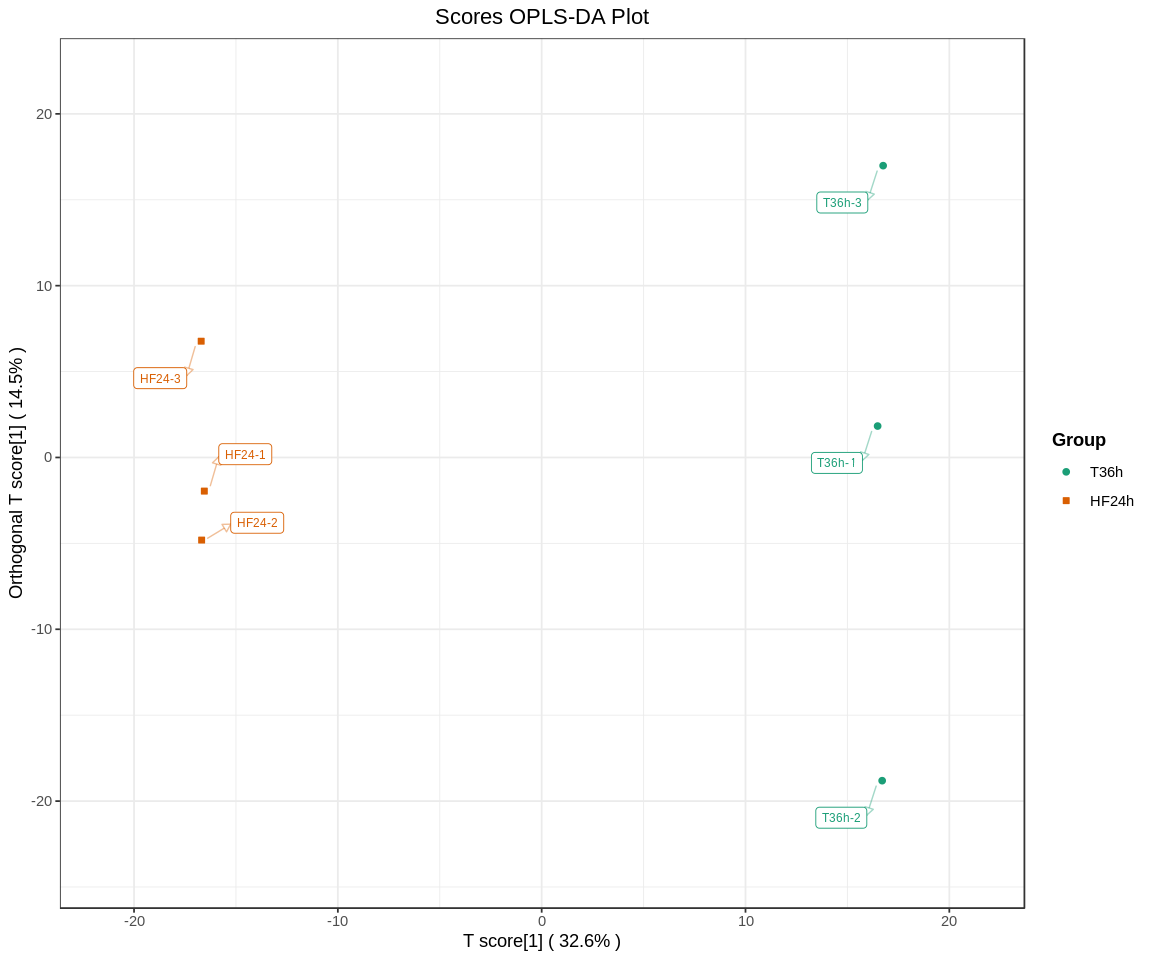

Supplement: Supplementary file 1 [file ijms-25-00310-s001.zip › Figure S1/T36HvsHF24H.png]

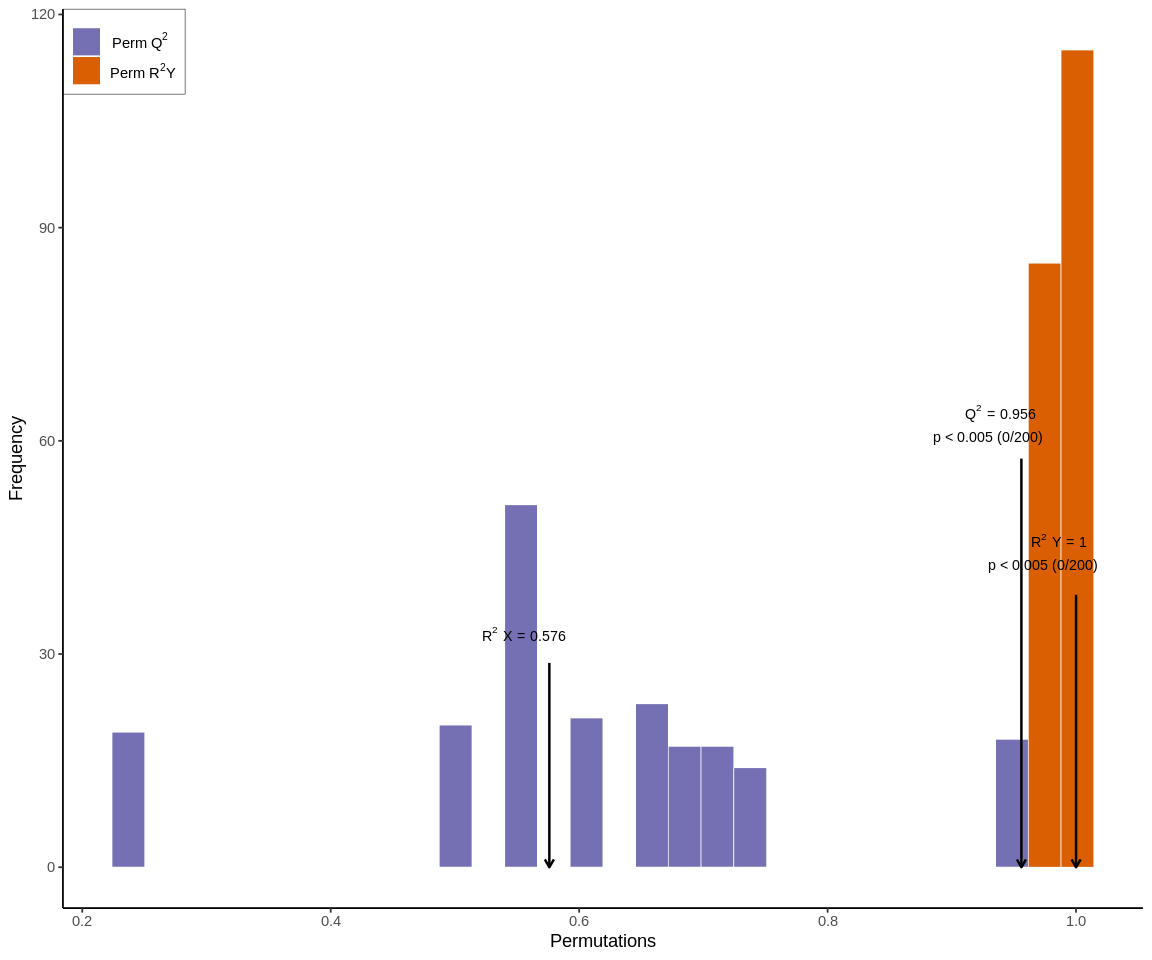

Supplement: Supplementary file 1 [file ijms-25-00310-s001.zip › Figure S2/CK_vs_HF24h_PLS-DA_permutation.png]

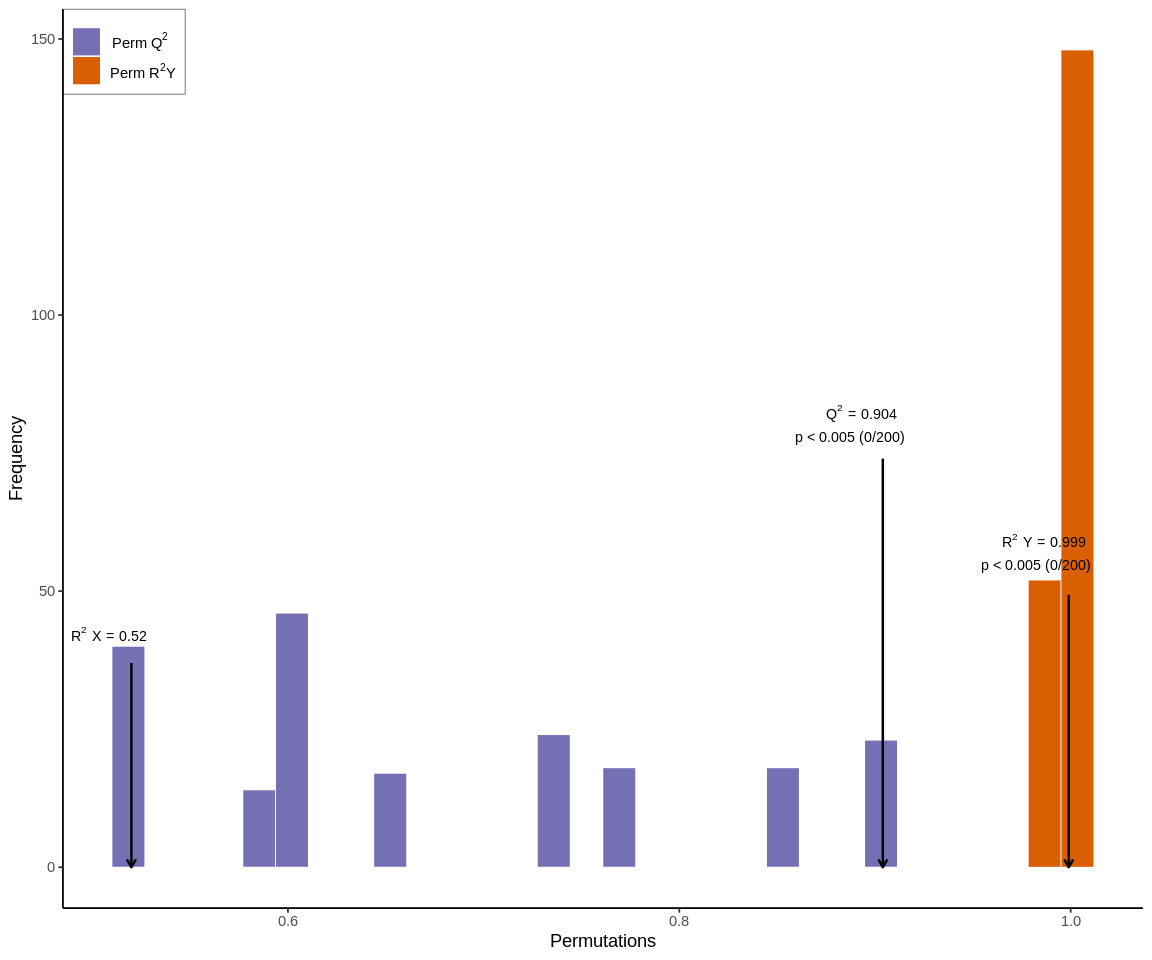

Supplement: Supplementary file 1 [file ijms-25-00310-s001.zip › Figure S2/CK_vs_T12h_PLS-DA_permutation.png]

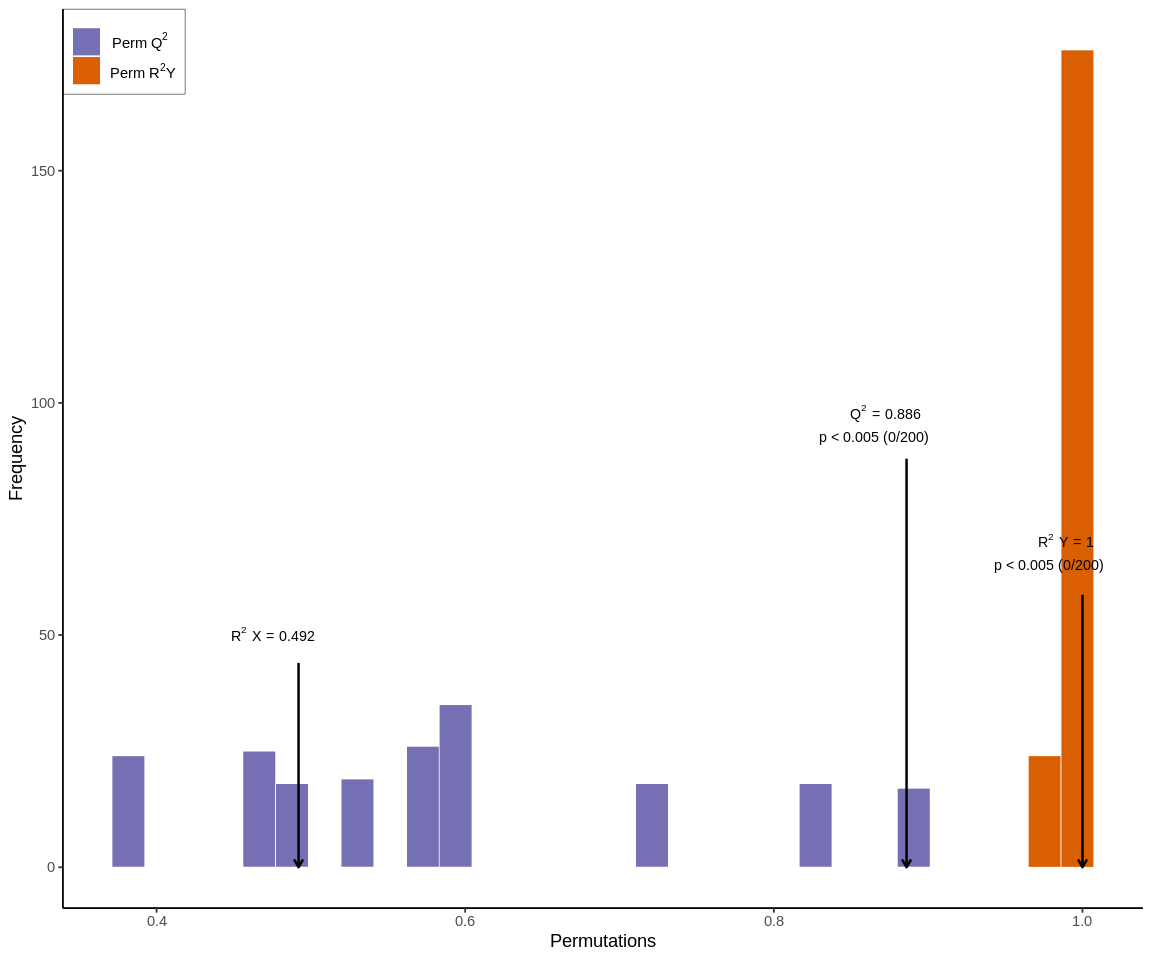

Supplement: Supplementary file 1 [file ijms-25-00310-s001.zip › Figure S2/CK_vs_T36h_PLS-DA_permutation.png]

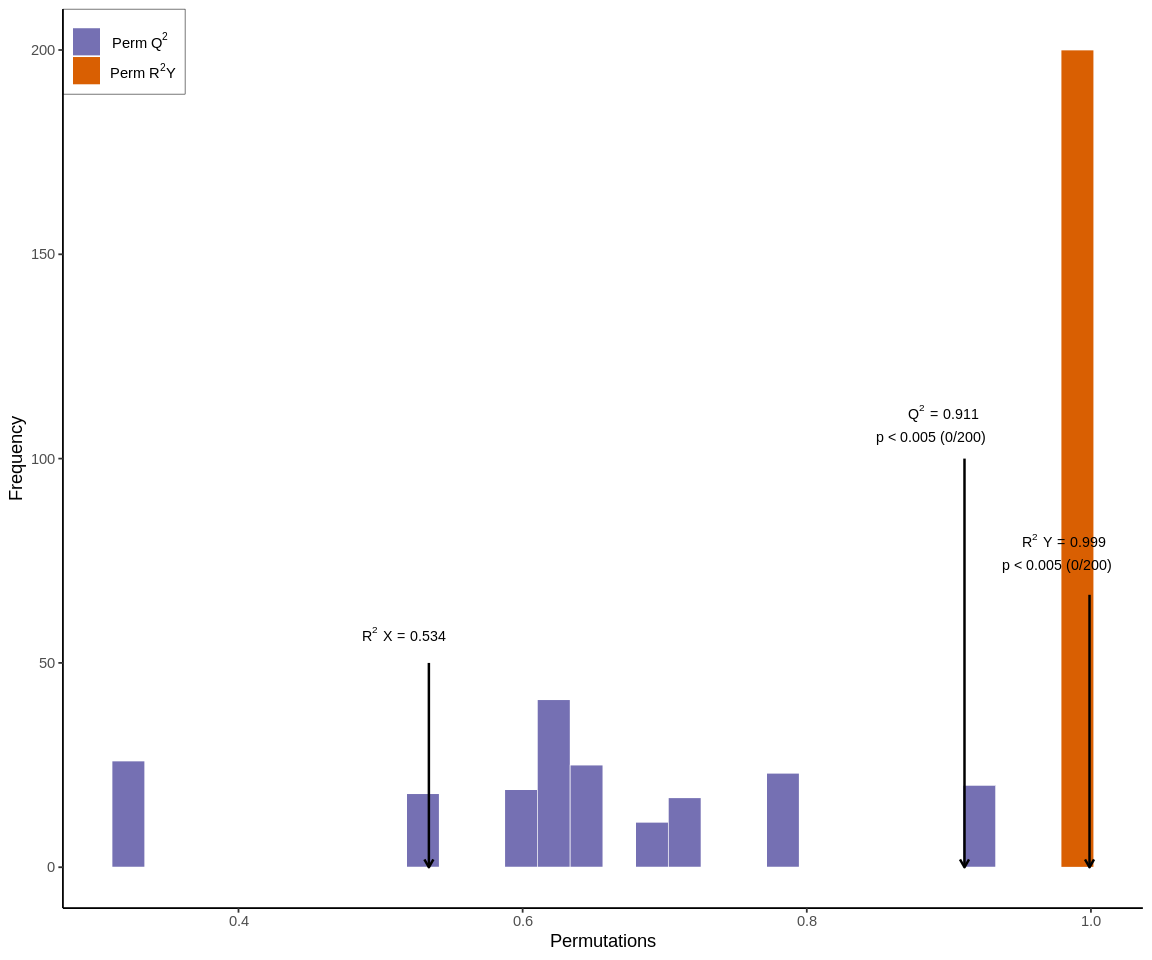

Supplement: Supplementary file 1 [file ijms-25-00310-s001.zip › Figure S2/T12h_vs_HF24h_PLS-DA_permutation.png]

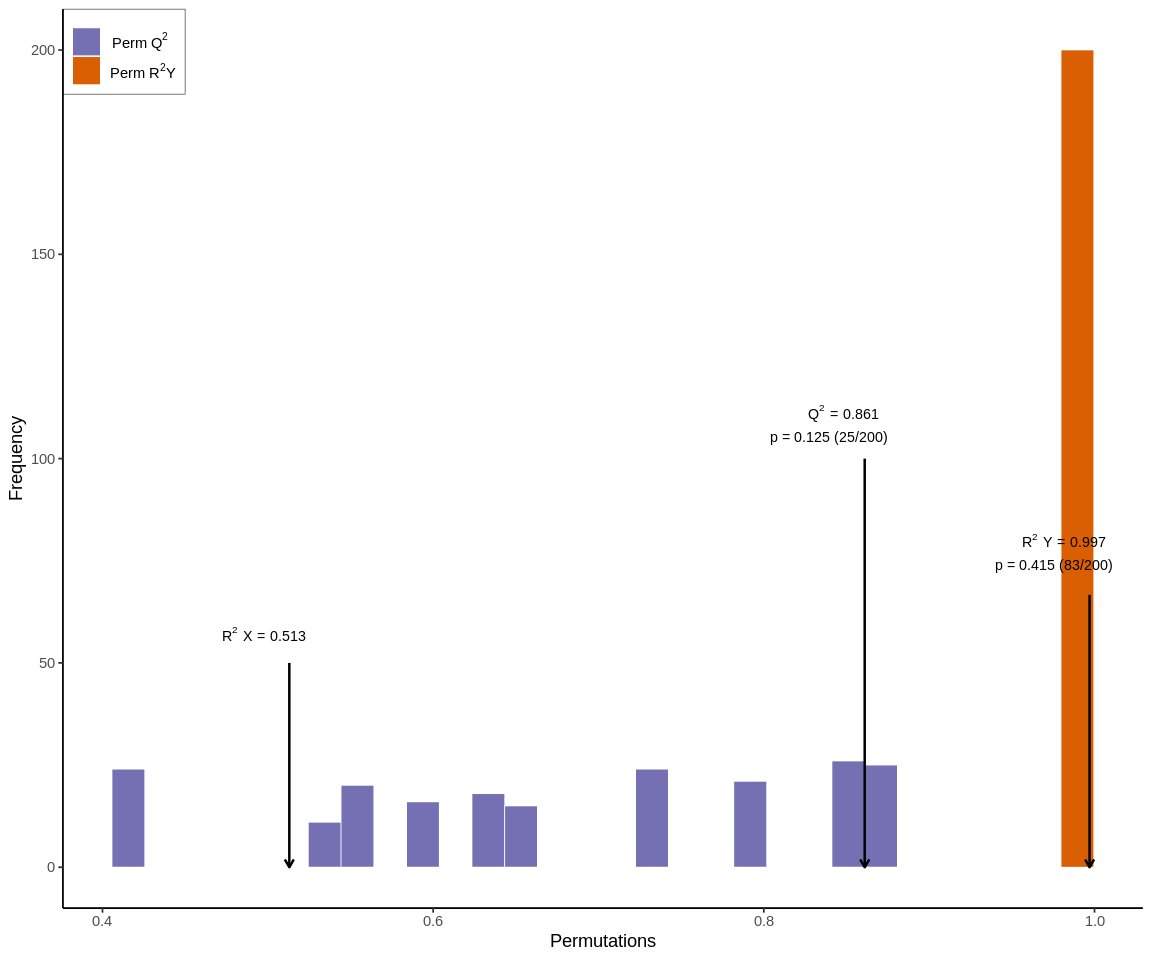

Supplement: Supplementary file 1 [file ijms-25-00310-s001.zip › Figure S2/T12h_vs_T36h_PLS-DA_permutation.png]

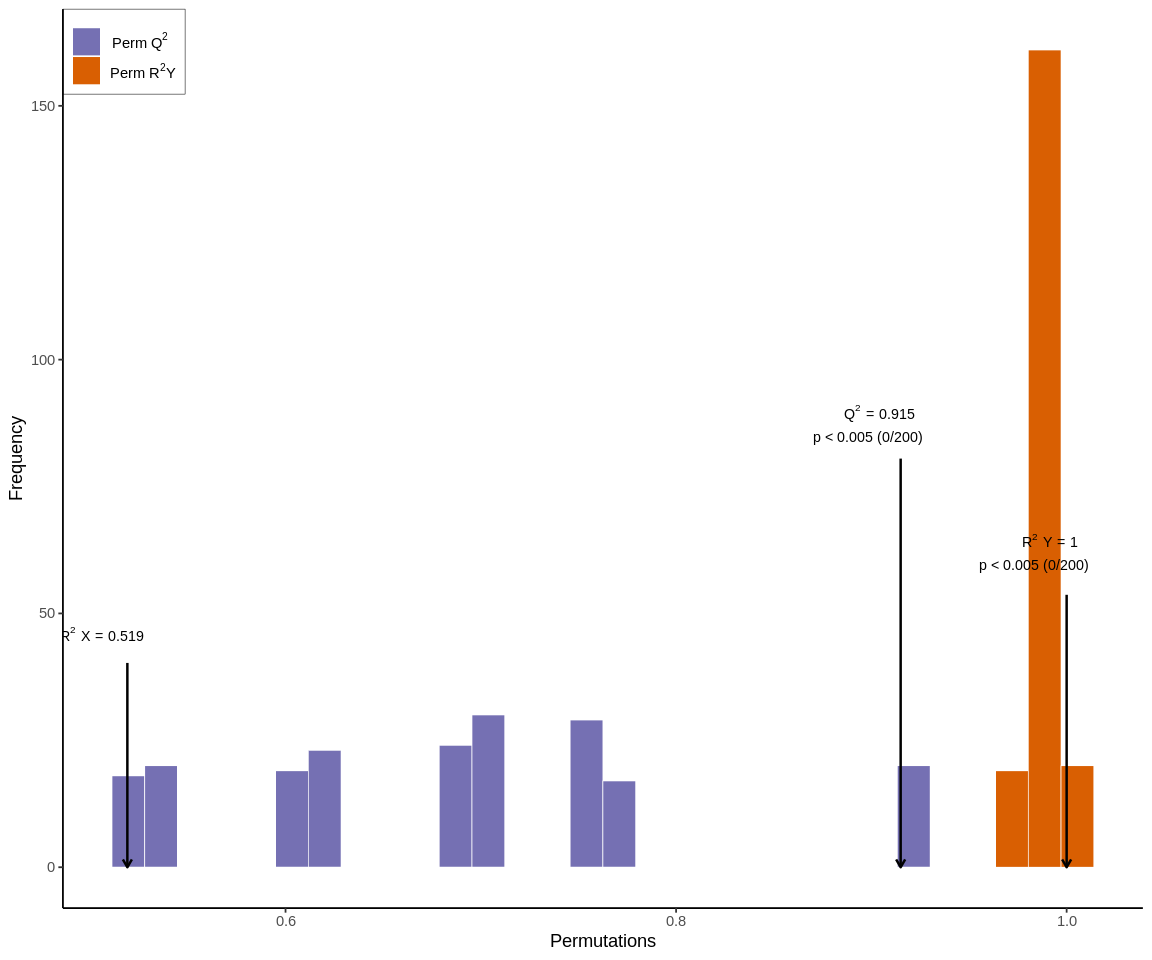

Supplement: Supplementary file 1 [file ijms-25-00310-s001.zip › Figure S2/T36h_vs_HF24h_PLS-DA_permutation.png]

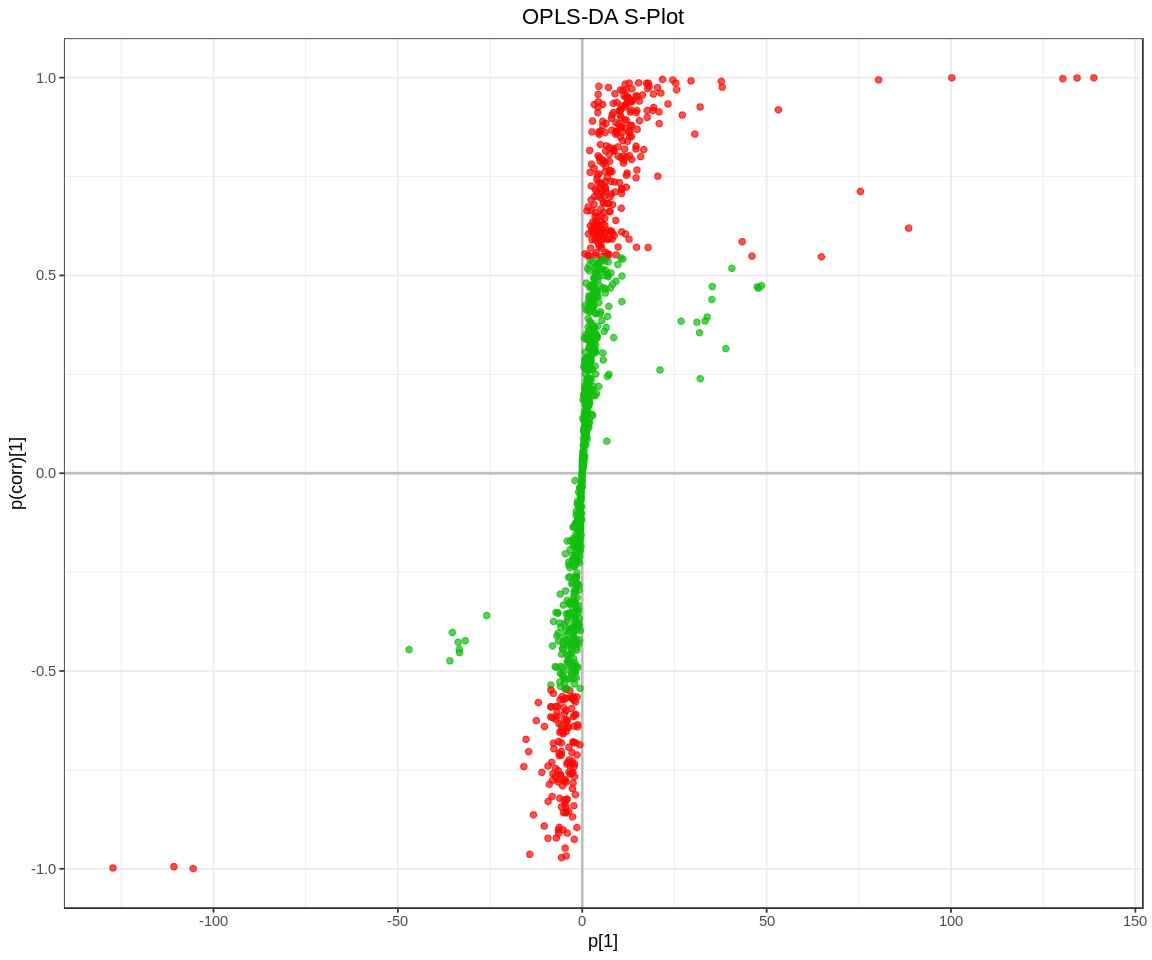

Supplement: Supplementary file 1 [file ijms-25-00310-s001.zip › Figure S3/CKvsHF24h.png]

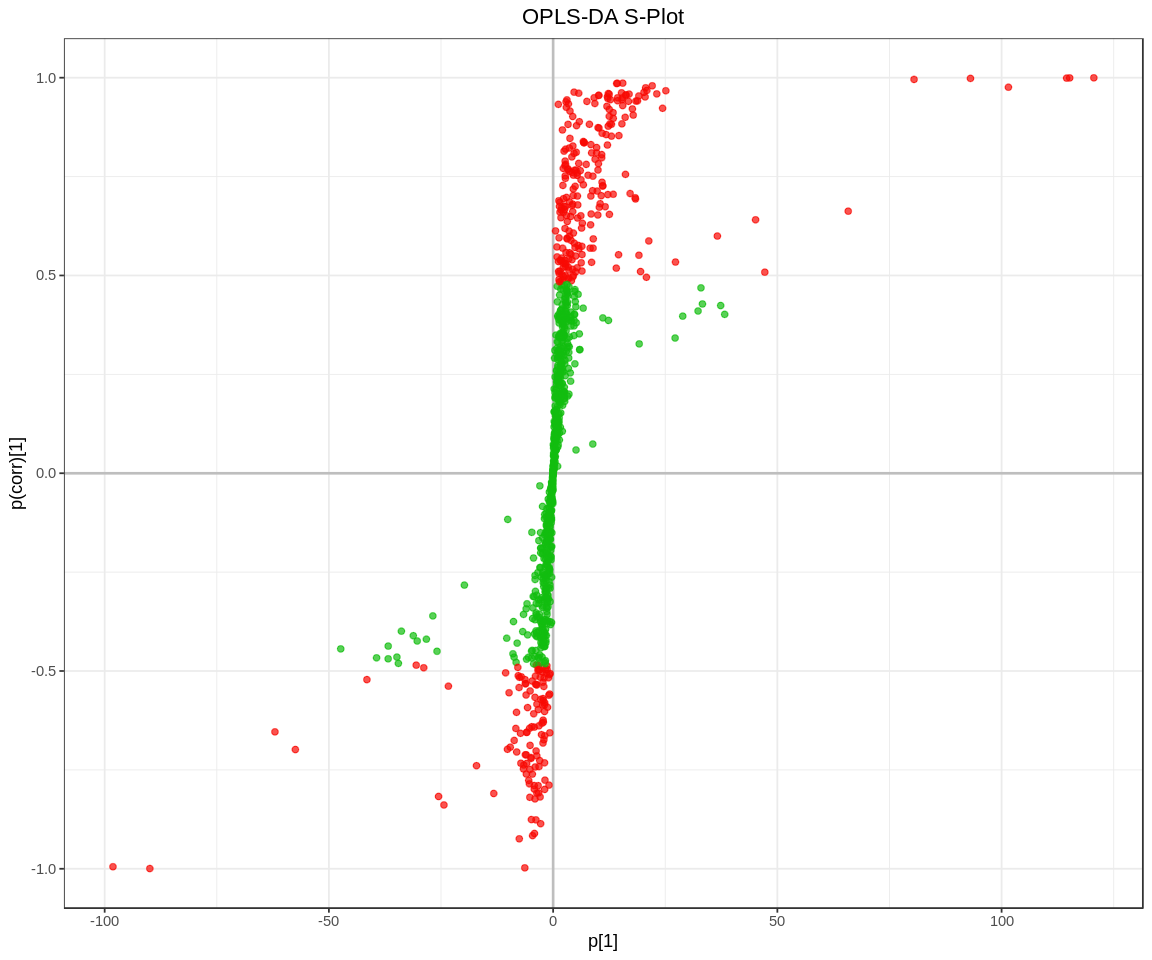

Supplement: Supplementary file 1 [file ijms-25-00310-s001.zip › Figure S3/CKvsT12h.png]

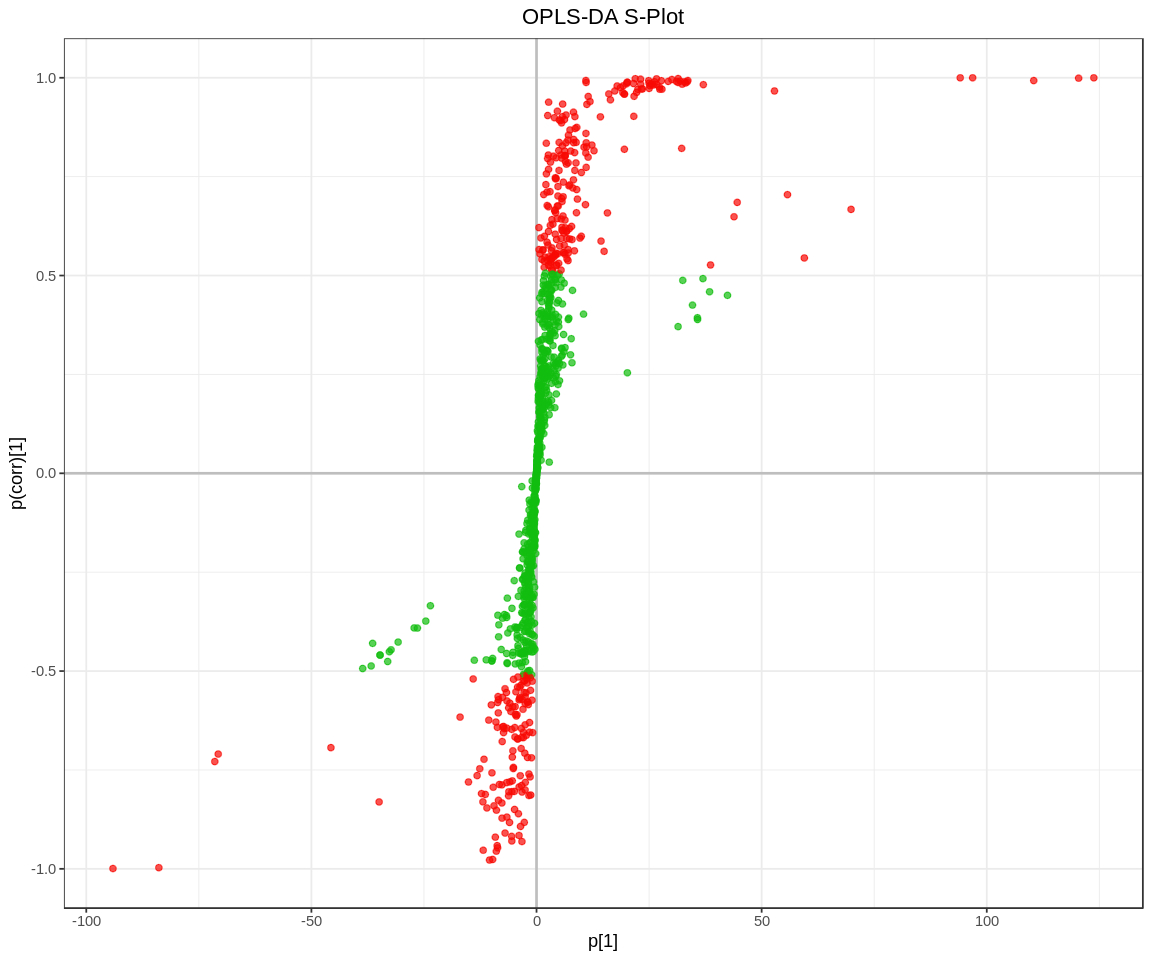

Supplement: Supplementary file 1 [file ijms-25-00310-s001.zip › Figure S3/CKvsT36h.png]

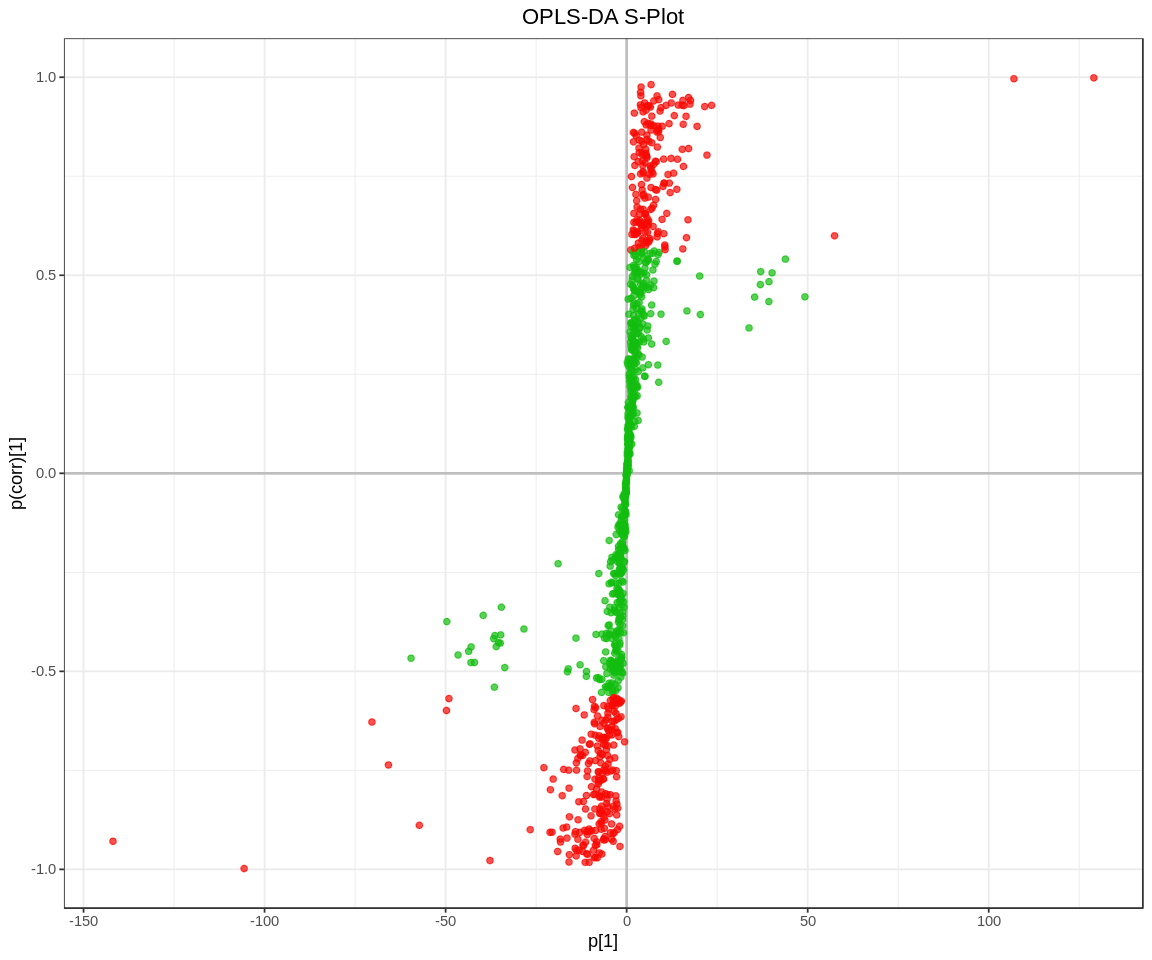

Supplement: Supplementary file 1 [file ijms-25-00310-s001.zip › Figure S3/T12hvsHF24h.png]

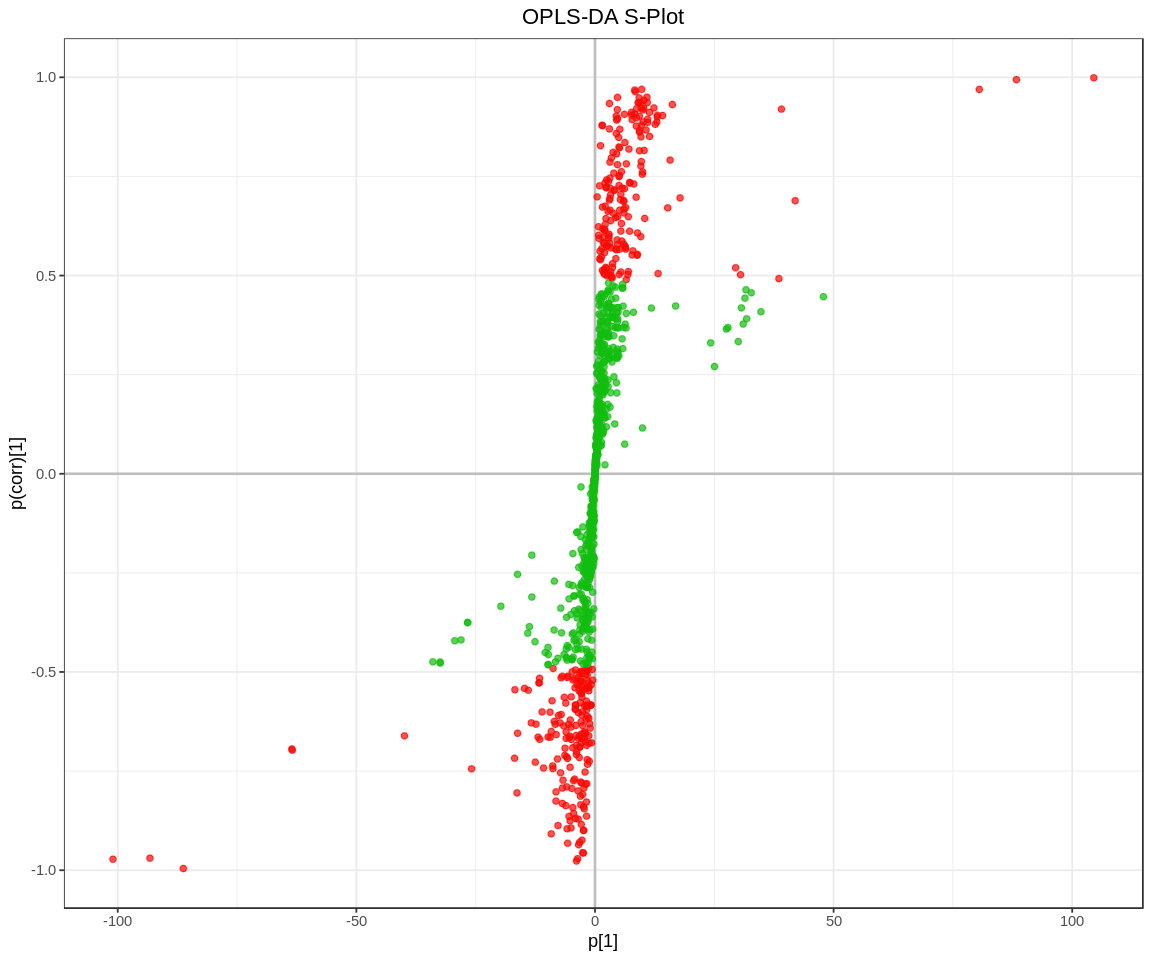

Supplement: Supplementary file 1 [file ijms-25-00310-s001.zip › Figure S3/T12hvsT36h.png]

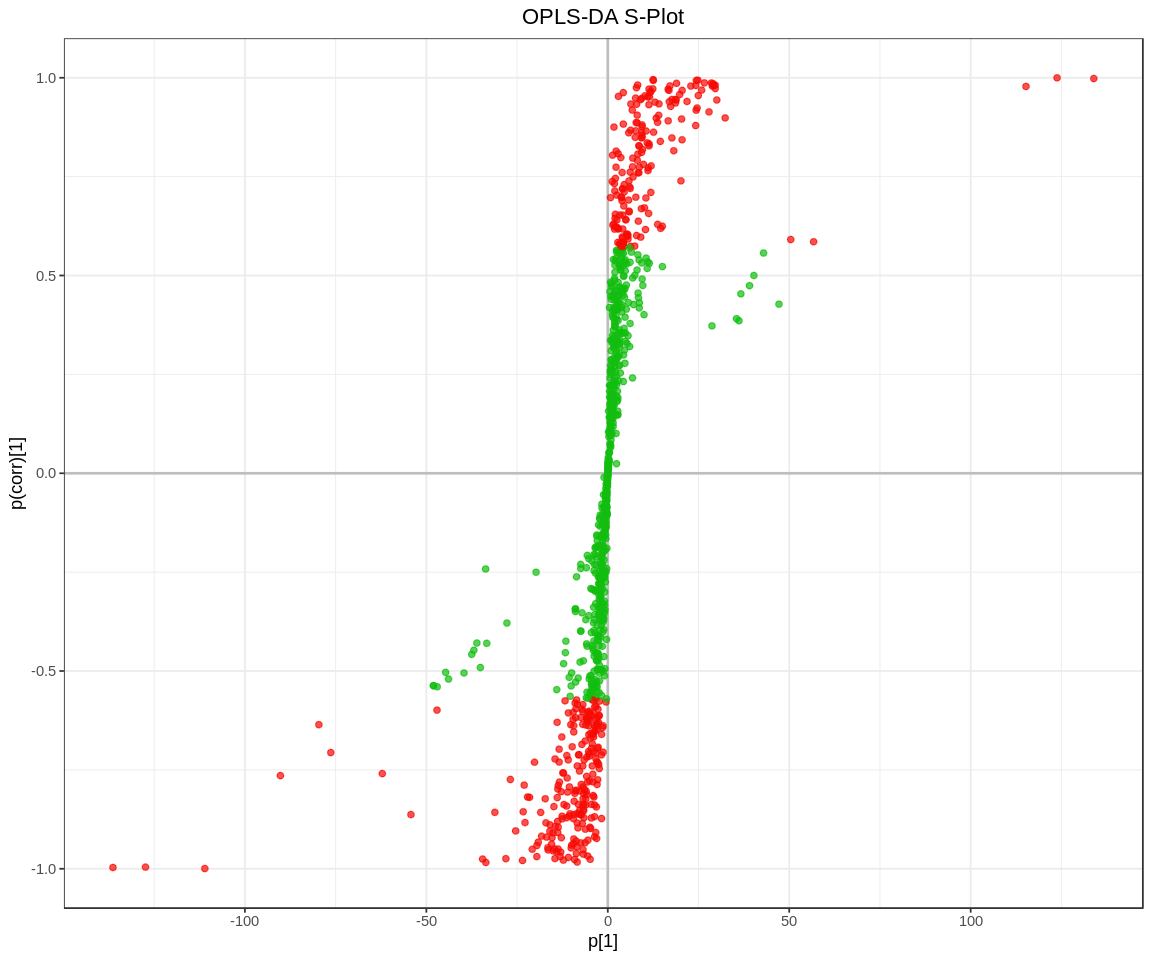

Supplement: Supplementary file 1 [file ijms-25-00310-s001.zip › Figure S3/T36hvsHF24h.png]
